# Multi-resistant gram negative enteric bacteria causing urinary tract infection among malnourished underfives admitted at a tertiary hospital, northwestern, Tanzania

**DOI:** 10.1186/s13052-015-0151-5

**Published:** 2015-06-19

**Authors:** Maimuna Ahmed, Nyambura Moremi, Mariam M. Mirambo, Adolfine Hokororo, Martha F. Mushi, Jeremiah Seni, Erasmus Kamugisha, Stephen E. Mshana

**Affiliations:** Department of Pediatrics and Child Health, Weill Bugando School of Medicine, Catholic University of health and allied sciences, Mwanza, Tanzania; Department of Microbiology and Immunology, Weill Bugando School of Medicine, Catholic University of health and allied sciences, P.O.Box 1464, Mwanza, Tanzania; Department of Biochemistry and Molecular Biology, Weill Bugando School of Medicine, Catholic University of health and allied sciences, Mwanza, Tanzania

**Keywords:** Malnourished, Underfives, UTI

## Abstract

**Background:**

Infections are common complications occurring in malnourished childrenas a result of impaired immunity. Urinary tract infections (UTI) have been found to be the commonest cause of fever in normal children in developing countries. However, data regarding UTI among malnourished children is limited because in most of time severe and moderately malnourished children are afebrile despite significant bacteriuria.

**Methods:**

A total of 402 malnourished underfives were enrolled. Demographic and other clinical characteristics were collected using standardized data collection tool. Urine specimens were cultured and interpreted according to standard operating procedures. Data were analyzed using STATA version 11.

**Results:**

Out of 402 malnourished underfives, 229 (56.9 %) were male. The median age in months was 17 (IQR; 12–31). Of 402 malnourished underfives, 83 (20.3 %) had significant bacteriuria of gram negative enteric bacteria. *Escherichia coli* 35/84 and *Klebsiella pneumonia* 20/84 were predominant bacteria isolated. More than 37 % of isolates were resistant to third generation cephalosporins with all of them exhibiting extended spectrum beta lactamase (ESBL) phenotype. Rates of resistance to ampicillin, amoxillin/clavulanic acid, gentamicin and ciprofloxacin were 82/84 (98.7 %), 47/55 (85.4 %), 45/84 (57.8 %) and 9/84 (10.8 %) respectively. Decrease in age and increase in lymphocytes count were independent factors on multivariate logistic regression analysis found to predict UTI (*p* < 0.05).

**Conclusions:**

Multi-resistant gram negative enteric bacteria are common cause of UTI among underfives. A significant number of severe and moderate malnourished children with bacteriuria had no fever. Therefore, routine testing for UTI is emphasized in all malnourished underfives so that appropriate treatment can be initiated.

## Background

UTI is among common infections in underfives, commonly presenting with fever and vomiting, whereby in malaria endemic area may be misdiagnosed and treated as malaria cases. Studies in this setting have demonstrated the high prevalence of UTI among febrile children with the prevalence of 39 % and above [1, 2]. The prevalence of UTI among malnourished underfives in Africa has been found to range from 11.3 % to 26.1 % [3, 4]. It has been observed that UTI can be a common complication among malnourished children [5, 6] and can further complicate the course of malnutrition because of associated poor feeding, diarrhea and vomiting [7]. Empirical management of UTI among malnourished underfives is a challenge because of lack of specific presentation features [5, 8]. Fever is not reliable asit is not common feature in severe and moderate malnourished underfives. In addition in developing countries fever can be due to other endemic diseases such as malaria [5]. Moreover, the diagnostic methods such as urine culture are not commonly available in most health facilities in developing countries.

Different pathogens have been found to cause UTI among underfives with majority of studies reporting *E. coli* to be apredominant pathogen [1, 2]. Also, *Staphylococcus aureus* strains have been reported in some studies as the common pathogens [9, 10]. Due to increase in the magnitude of antibiotic resistance there is a need to routinely investigate specific pathogen causing UTI in every case so that effective antibiotic treatment can be used. However this can be difficult in developing countries and especially among malnourished underfives. Therefore these data are useful to clinicians in developing countries especially where culture is not available so that appropriate empirical treatment can be initiated timely.

## Methods

### Study design and study area

A hospital based cross sectional study was conducted from September 2012 to January 2013 at the Bugando Medical Centre (BMC) in Mwanza, Tanzania. BMC is a tertiary, consultant and teaching hospital serving a population of about 13 million from the lake zone of Tanzania.

### Inclusion and exclusion criteria

All malnourished children by WHO criteria [11] whom parents/guardians/care-givers willingly signed a written informed consent for their children to participate into the study were included. Children with congenital malformation, with catheter and with neurological defects were excluded.

### Sample size and sampling procedure

Minimum sample size of 384 malnourished children was estimated using Kish Lisle formula for cross-sectional studies. Allmalnourished children aged 6–60 months admitted to pediatric wardswere conveniently sampled until a desired sample size was reached. A total of 402 malnourished children who met the inclusion criteria were enrolled. Demographic data and other related factors related to malnutrition and urinary tract infections were collected using a standardized data collection tool. Nutritional status were measured as described by WHO guidelines [11] in which measurements of weight for length or height were interpreted using Z- score for mild (−1SD), moderate (−2SD) and severe (−3SD) malnutrition.

### Samplecollection and laboratory analysis

For children above two years of age mid stream urine (MSU) was obtained following standard procedures [12, 13] and for children 6 months to 2 years, suprapubic urine was obtained aseptically. All specimens were transported to the microbiology laboratory within one hour of collection for prompt processing.

A definitive culture for MSU and suprapubic urine was done on cysteine lactose electrolyte deficient Agar (CLED), MacConkey agar and blood Agar plates (Oxoid, UK). Plates were incubated at 37 °C and read after 24 h. A diagnosis of UTI was made when at least 10^5^ colony forming unit (CFU)/ml of MSU and any colony count for suprapubic urine were detected [14]. High colony counts with more than one species of bacteria were considered as contamination and culture was repeated. Identification of the bacterial isolates was done using in-house biochemical techniques [15].

Drug susceptibility testing was performed on pure coloniesusing disk diffusion method according to the Clinical and Laboratory Standard Institute CLSI [16]. Antibiotic discs tested were ampicillin (10 μg), amoxicillin/clavulanic acid (20/10 μg), ciprofloxacin (5 μg), gentamicin (10 μg), ceftriaxone (30 μg), ceftazidime (30 μg) and ertapenem (10 μg) (Oxoid, UK). *Escherichia coli* ATCC 25922 and *Staphylococcus aureus* ATCC 25923 were used for quality control of all microbiological tests.

In addition, about 2 ml of blood in EDTA container (BD Vacutainer, Nairobi, Kenya) was collected for WBC and lymphocyte count and estimated using hematological analyzer (Beckman coulter (UK) LTD).

### Statistical analysis

Data were entered into a computer using Microsoft Excel 2007, cleaned and analyzed using STATA version 11 (college station, Texas). Fisher exact and Chi-square tests were done to establish statistical difference in proportions for categorical data. Predictors of positive urine culture were determined by univariate and multivariate logistic regression analysis. Statistical significance was set at *P* < 0.05 with confidence interval of 95 %.

### Ethical consideration

The research proposal was approved by the Joint BMC-CUHASethical review board ethics. Informed consent was obtained from parents or guardians before children were enrolled.

## Results

### Demographic characteristics

A total of 402 malnourished children were enrolled into the study between September and January 2013. Of 402 children, 229 (56.9 %) were female. The median age was 17 (IQR; 12–31) months. Majority of children 54 % (217/402) had axillary temperature below 37.5 °C. Only 31 (7.7 %) children tested positive for HIV (Table 1).

**Table 1:** Demographic characteristics of 402 malnourished children

Variable	*N* %, median
Age	17 (IQR: 12–31)
Sex	
Male	173 (43 %)
Female	229 (56.9 %)
Fever	
Temp < 37.5	217 (53.98 %)
Temp > 37.5	185 (46.02 %)
White blood cell count	9400 (IQR:6700–13000)
Neutrophils %	46.7 % ± 23.1
Lymphocyte %	39.6 % ± 18.31
HIV	
Reactive Non- reactive	31 (7.7 %)
Non- reactive	371 (92.3 %)

### Isolates and susceptibility results

Out 402 children; 84 (20.65 %) had significant bacteriuria of gram negative enteric bacteria. *Escherichia coli* 35/84 (41.2 %) formed majority of the isolates. Other bacteria isolated were *Klebsiella pneumoniae* 20/84 (23.8 %) and other enteric gram negative (*Proteus* spp., *Enterobacter* spp., *Citrobacter* spp., and *Serratia* spp.) 29/84 (34.5 %) Table 2.

**Table 2:** Rates of resistance of 84 isolates from urine of malnourished children

Pathogen	AMP	CN	CIP	AMC	CRO	CAZ	ERT
*E. coli* (35)	33 (97.1 %)	15 (42.8 %)	5 (14.3 %)	30 (85.7 %)	12 (34.3 %)	14 (40 %)	1 (2.86 %)
*K. pneumoniae* (20)	20 (100 %)	14 (70 %)	3 15.0 %)	17 (85.0 %)	10 (50.0 %)	12 (60 %)	0 (0.0 %)
^a^Other gram negative (29)	29 (100 %)	19 (65.5 %)	1 (3.4 %)	ND	15 (51.7 %)	15 (51.7 %)	(0.0 %)
Total (84)	82 (98.7 %)	48 (57.8 %)	9 (10.8 %)	47 (85.4 %)	37 (44.0 %)	41 (48.8 %)	1 (1.2 %)

*AMP* Ampicilin, *CN* Gentamicin, *CIP* Ciprofloxacin, *AMC* Amoxicillin/Clavulanic acid, *CRO* Ceftriaxone, *CAZ* Ceftazidime, *ERT* Ertapenem

^a^Other gram negative (Proteus spp., Enterobacter spp., Citrobacter spp., Serratia spp.,)

Out of 84 isolates tested; 37 (44 %) and 41 (48.8 %) were resistant to ceftazidime and ceftriaxone respectively; with all of them exhibiting extended spectrum beta lactamases (ESBL) phenotype. The rates of resistance to ampicillin, amoxillin/clavulanic acid, gentamicin and ciprofloxacin were 82/84 (98.7 %), 47/55 (85.4 %), 45/84 (57.8 %) and 9/84 (10.8 %) respectively. One isolate was found to be resistant to ertapenem (Table 2).

### Predictors of significant bacteriuria

In this study it was observed that as age increases number of children with significant bacteriuria decreases (OR 0.978, 95 % CI; 0.95-0.99), *p* = 0.038). Also it was noted that increase in lymphocyte predicts significant bacteriuria (OR 1.01, 95 % CI; 1.00-1.03), *p* = 0.038). Female children had 1.23 times risk of having significant bacteriuria however the difference was not statistically significant (*p* = 0.467, 95 % CI; 0.7-2.16). Of 217 children without fever; 49 (22.6 %) had significant bacteriuria compared to 34 (18.4 %) of children with fever (*p* = 0.987) Table 3.

**Table 3:** Factors associated with urinary tract infection in malnourished children

Characteristics	Culture + (N, %)	Univariate		Multivariate	
		OR (95 % CI)	*P* value	OR (95 % CI)	*P* value
Age (years)^a^	18 (IQR 12–24)	0.984 (0.96-1.0)	0.066	0.978 (0.95-0.99)	0.038
Sex					
Male (173)	34 (19.6 %)				
Female (229)	49 (21.4 %)	1.11 (0.681-1.81)	0.681	1.23 (0.70-2.16)	0.467
Fever					
No (217)	49 (22.6 %)	1			
Yes (185)	34 (18.4 %)	0.77 (0.47-1.25)	0.300	0.99 (0.56-1.74)	0.987
WBC^b^	12.8 ± 12	1.00 (0.987-1.03)	0.041	1.00 (0.98-1.03)	0.445
Lymphocytes	44 ± 18.6	1.01 (1.00-1.03)	0.023	1.01 (1.00-1.03)	0.038
HIV					
Negative (371)	77 (20.75 %)	1			
Positive (31)	6 (19.35 %)	1.09 (0.43-2.7)	0.853	0.95 (0.36-2.49)	0.922
Nutritional status					
Mild 147	21 (14.3 %)	1			
Moderate 77	14 (18.2 %)	1.3 (0.58-2.9)	0.456		
Severe 178	48 (27.0 %)	2.2 (1.2- 4.1)	0.005	1.28 (0.93-1.77)	0.126

^a^Median age

^b^Mean white blood cell counts(WBC)

On univariate analysis children with severe malnutrition 27 % (48/178) had significant more bacteriuria than children with moderate malnutrition 18.2 % (14/77) and mild malnutrition14.3 % (21/147) (*p* = 0.016).

Of 178 severe malnourished children, only 62 (34.8 %) had fever compared to 87 (57.8 %) and 38 (49.3 %) of children with mild and moderate malnutrition respectively (*p* < 0.001).

## Discussion

This study investigated 402 malnourished under-fives attending Bugando Medical Centre with median age of 17 months. As observed previously in the same setting majority of children were below 24 months [1]. The prevalence of significant bacteriuria in this study was 20.6 %. The observed prevalence is similar to what was observed by Msaki et al. [2] among under-fives from the general population in Mwanza city. However, the observed prevalence is significantly lower (*p* < 0.001) than reported prevalence among febrile children admitted in the same hospital two years ago [1]. This could be explained by the fact that the participants enrolled in previous study had fever whereas in this study fever was not used as an inclusion criterion. Fever in malnourished children has been found to be associated with significant bacteriuria [5].

As observed in previous study in the same setting [1, 2] children below 2 years had 1.5 times more risk of significant bacteriuria than children above two years. This confirms what has been explained previously that poor hygienic practices in children below two years contribute significantly to acquisition of UTI [17, 18]. Also in this study female sex had 11 % increase risk of significant bacteriuria compared to male sex. This has been observed in several previous studies as a risk of UTI [1, 19, 20]. Lymphocytosis in the current study was another factor which was found to predict significant bacteriuria independently. This may be due to the fact that lymphocytosis has been observed to be a common phenomenonin malnourished children [21].

In the present study fever was not a predictor of significant bacteriuria as observed in the previous study [5]. It has been documented that malnourished children can have infection without fever [22]. This is due to immunocompromised state which impairs ability to mount innate immune responses against infections [23] hence asymptomatic bacteriuria is common in this population. Further analysis in this study confirmedthis whereby children with mild and moderate malnutrion significantly presented with fever than those severely malnourished (*p* < 0.001).

In the current study all isolates were gram negative enteric bacteria where by *Escherichia coli* strainswere predominant isolates. *E.coli* has been found to be the commonest cause of UTI in various populations; this is due to ascending nature of the pathogenesis of UTI [10]. Majority of these isolates were multi-drug resistant with more than 97 % being resistant to ampicillin which isthe commonest antibiotics usedasfirst line treatment in most health facilities in Tanzania. Similar findings have been observed in previous studies [24–26] in same setting among isolates from wounds and blood stream infections.

The thirdline antibiotic treatment in Tanzania is third generation cephalosporins; which in this study more than 44 % of the isolates were found to be resistant to this class of antibiotic. Compared to a study by Festo et al.*,* which was done 2 years earlier in the same setting and wards, there is fourfold increase of resistance of *E.coli* to third generation cephalosporins. Cephalosporinsare overused in this setting; this may be the most probable reason for the resistance increase. Nevertheless, the resistance rate of *E.coli* to ciprofloxacin has remained stable for the past three years; this is due to the fact that ciprofloxacin is not commonly used in children in this setting.

## Conclusion

Gram negative enteric bacteria resistant to ampicillin, gentamicin, and amoxicillin/clavulanic acid and third generation cephalosporins are common cause of significant bacteriuria among malnourished underfives. Based on these results the empirical treatment using ciprofloxacin could be employed in our setting; however ciprofloxacin is not recommended in children unless in serious life threatened infection. Therefore, to avoid complications associated with significant bacteriuria, routine testing for UTI and susceptibility testing in all malnourished underfives should be emphasized so that appropriate treatment can be initiated timely.
